# Quality of life, functional outcome, and voice handicap index in partial laryngectomy patients for early glottic cancer

**DOI:** 10.1186/1472-6815-5-3

**Published:** 2005-05-12

**Authors:** Tolga Kandogan, Aylin Sanal

**Affiliations:** 1 Department of Otolaryngology & Head-Neck Surgery, SSK Izmir Hospital, Izmir, Turkey; 2 Selen Ses Merkezi, Ali Cetinkaya Bulvari No:31/1 Daire 24 Alsancak Izmir 35220 Turkey

## Abstract

**Background:**

In this study, we aim to gather information about the quality of life issues, functional outcomes and voice problems facing early glottic cancer patients treated with the surgical techniques such as laryngofissure cordectomy, fronto-lateral laryngectomy, or cricohyoidopexi. In particular, consistency of life and voice quality issues with the laryngeal tissue excised during surgery is examined. In addition, the effects of arytenoidectomy to the life and voice quality are also studied.

**Methods:**

29 male patients were enrolled voluntarily in the study. The average age was 53.9 years. Three out of 10 patients with laryngofissure cordectomy also had arytenoidectomy. 11 patients had fronto-lateral laryngectomy with Tucker reconstruction, two of which also had arytenoidectomy. There were eight patients with cricohyoidopexi and bilateral functional neck dissection. Three of these patients also had arytenoidectomy. In bilateral functional neck dissection cases, spinal accessory nerve was preserved and level V of the neck was not dissected. None of the patients had neither radiotherapy nor voice therapy. Cordectomy patients never had a temporary tracheotomy or were connected to a feeding tube. Data was collected for 13 months for the cordectomy group, 14 months for fronto-lateral laryngectomy and cricohyoidopexi groups on average post-operatively. Statistical analysis in this study was carried out using the one-way analysis of variance, and the Post-Hoc group comparisons were made after Bonferroni and Scheffé-procedures.

In order to determine the effects of arytenoidectomy, a regression analysis is carried out to see if there are statistical differences in answers given to the survey questions among patients who were arytenoidectomized during their surgeries.

**Results:**

There was a statistically significant difference between cordectomy and cricohyoidopexi group in answers to the University of Washington- Quality of Life- Revised survey part 1. (p = 0). A statistically significant difference was also established between cordectomy and fronto-lateral laryngectomy groups, as well as between cordectomy and cricohyoidopexi groups in answers to the University of Washington- Quality of Life- Revised survey part 2. (p = 0,036 and p = 0.009, respectively). Cricohyoidopexi group has given the lowest scores and the cordectomy group has given the highest scores in three survey questions representing the quality of life, performances and new voices. These ranges are also consistent with the laryngeal tissue excised during surgery (cricohyoidopexi > fronto-lateral laryngectomy > cordectomy). There was no statistically significant difference between groups in Performance Status Scale for Head and Neck cancer patients instrument. The difference between the Voice Handicap Index and Voice Handicap Index (functional); Voice Handicap Index (physical) and Voice Handicap Index (emotional) scores in three patient groups was not significant either. All of the patients evaluated that their new voices have similar functional, physical and emotional impact on their life. Decanulation and oral feeding times of cricohyoidopexi and fronto-lateral laryngectomy patients are found to be significantly longer than cordectomy patients. Lastly, the removal of arytenoid does not have any significant adverse effects on the quality of life, the functional outcomes, or the quality of voice.

**Conclusion:**

In the present study, all patients with early glottic cancer, treated with different surgical technics reported fairly good quality of life outcomes, functional results and voice qualities. This study also finds that the removal of arytenoid does not have any adverse effects on the quality of life and voice from the patients' point of view.

## Background

In clinical research, QOL is recognized as an important endpoint in addition to the traditional endpoints such as response rate, disease-free survival, and overall survival [1]. It is particularly relevant for patients with head and neck cancer because social interaction and emotional expression depend to a great extent on the structural and functional integrity of the head and neck region [2]. Today, treatment policies are aimed to improve and maintain QOL during and after treatment.

Although early-stage glottic carcinomas (T1 and T2) are currently treated with radiation therapy or endoscopic laser resection with success, conservation laryngeal surgeries such as laryngofissure [C], [FLL], or [CHP] are also available alternatives for treatment [3]. In the surgical management of laryngeal malignancies, surgical alteration or removal of the larynx affect some of the most fundamental life functions including airway, digestion, and speech.

The measurement of voice quality outcomes following treatment for laryngeal cancer is a relatively new concept. Voice quality encompasses social, psychosocial, mental and physical components. Given patients with same diagnosis and same treatment, the outcome data may be totally different for them. Evaluation of voice quality following laryngeal surgery can be one of the first attempts to assess the functional outcome following successful treatment.

Although health-related quality of life issues in early stages of laryngeal cancer are more important when comparing different therapy modalities such as surgery versus radiation therapy, in this study we aim to gather information about the QOL issues, functional outcomes and voice problems facing early glottic cancer patients that were treated with the surgical techniques mentioned above.

Results of answers provided to survey questions are compared to determine whether life and voice quality issues are consistent with the laryngeal tissue excised during surgery. In addition, the effects of arytenoidectomy to the life and voice quality are also studied.

## Methods

29 male patients were enrolled voluntarily in the study. The average age was 53.9 years. There were 10 patients with laryngofissure [C], three of which also had (A). Two out of 11 patients with [FLL] with Tucker reconstruction also had (A). There were eight patients with [CHP] and (BFND). Three of these patients also had (A). In BFND cases, spinal accessory nerve was preserved and level V of the neck was not dissected. Margins were checked with frozen sections per-operatively. None of the patients had neither radiotherapy nor voice therapy. [C] patients never had a temporary tracheotomy nor connected to a feeding tube. Data was collected in [C] group over 13 months, [FLL] group over 14 months and in [CHP] group over 14 months on average post-operatively. The data on the stage of the laryngeal cancer, the decanulation and the oral feeding times, the surgeries applied, and the scores of the questionnaires are listed in additional file 1. [see Additional file 1]

### UW-QOL-R

The UW-QOL-R was given to all patients treated for early glottic cancer with the above-mentioned surgeries. The patient selects an answer from three to five choices, depending on the question. The UW-QOL-R data were scored on a scale of 0 to 100, with a score of 100 being totally functional and 0 being completely incapacitated [4]. Two small modifications have been made to increase the sensitivity to the UW-QOL-R. First, "My speech is not normal, but I can say all words" was added to the speech domain. Second modification was the addition of a question regarding the use of saliva substitutes under the saliva domain [4].

In part 2 of UW-QOL-R, the patient answers to global quality of life questions, selects an answer from five to six choices, ranging from very poor to outstanding, or from much worse to much better, depending on the question.

### PSS-HN

In PSS-HN, eating in public (EIP), understandability of speech (UOS), and normalcy of diet (NOD) are each individually assessed on a zero to 100-point scale [4].

### VHI

The patients were instructed that these statements are how many people describe their voices and the effects of their voices on their lives. The patients marked the response that indicates how frequently they have the same experience. 0 = Never 1 = Almost Never 2 = Sometimes 3 = Almost Always 4 = Always.

0 to 30 = These are low scores, and indicate that most likely there is a minimal amount of handicap associated with the voice disorder. 31 to 60= Denotes a moderate amount of handicap due to the voice problem. 60 to 120= These scores represent a significant and serious amount of handicap due to a voice problem[5].

### Statistical Analysis

Statistical analysis in this study includes one-way analysis of variance, ANOVA, and the Post-Hoc group comparisons after Bonferroni and Scheffé-procedures.

In order to determine the effects of arytenoidectomy, the following regression model is adopted to see if there are statistical differences in answers given to survey questions among different patient groups that were arytenoidectomized during their surgeries.

Y = α + β_1_.CHP + β_2_.FLL + β_3_.A + β_4_.CHP.A + β_5_.FLL.A

where Y is the answer given by a patient to a question, α is the mean of answers given by patients who had the [C] surgery technique. [CHP] and [FLL] are dummy variables for surgery techniques [CHP] and [FLL], respectively. The coefficients of these variables will show the amount of difference in answers given by patients who had these techniques, and those who had the [C] technique. The t statistic of these coefficients will be used in testing whether these differences are statistically significant or not. Similarly, [A] is a dummy variable for patients who had the A procedure applied after these above-mentioned surgery techniques. Its coefficient quantifies the difference in answers given by patients who had A procedure applied after a surgery technique, and those who did not have. The last two variables are interaction variables between the [CHP] and [FLL] dummy variables, and the [A] dummy variable. Their purpose in the model is to test whether [A] applied after [CHP] or [FLL] leads to statistically different answers by patients than [A] applied after [C].

## Results

The regression results are given in Table 1. The effects of the [A] procedure were different depending on the previous surgical technique applied. If applied after [CHP], the answers of the patients to first questionnaire were generally higher, but insignificant than those of patients who had [A] after [C]. The measures of the second and third questions were higher too, if [A] is applied after [CHP], rather than after [C]. These measures are also statistically higher if [A] is applied after [FLL], rather than after [C], as well. However, the answers to first questionnaire are sometimes higher, sometimes lower if [A] is applied after [FLL], rather than after [C].

The mean values for each analyzed parameter in questionnaires for three patient groups are shown on table 2. There was a statistically significant difference between [C] and [CHP] group in answers to the UW-QOL-R questionnaire part 1. (p = 0). There was also a statistically significant difference between [C] and [FLL] group and [C] and [CHP] group in answers to the UW-QOL-R questionnaire part 2. (p = 0,036 and p = 0.009). [CHP] group have given the lowest scores and the [C] group has given the highest scores in three questionnaires to represent their quality of life, performances and new voices. These ranges are also consistent with the laryngeal tissue excised during surgery ([CHP]> [FLL]> [C]).

There was not a statistically significant difference between groups in PSS-HN instrument. The difference between the VHI and VHI-F, VHI-P, VHI-E scores in three patient groups were not statistically different either (Table 3). All of the patients evaluated that their new voices had similar functional, physical and emotional impact on their life. Decanulation and oral feeding times of [CHP] and [FLL] patients has been found to be significantly longer than [C] patients (Table 4).

## Discussion

Patients generally differ in their response to cancer and to the therapeutic interventions used in its treatment. Even patients with a similar oncologic site and stage who receive identical treatment can differ in their own assessment of quality of life [6].

Not surprisingly, [CHP] group have given the lowest scores and the [C] group has given the highest scores in three questionnaires, representing their quality of life, performances and new voices (Table 2). These ranges are also consistent with the laryngeal tissue excised during surgery ([CHP]> [FLL]> [C]). A possible reason for this finding could be that these [C] patients were never tracheotomized, so they were more comfortable in the postoperative period. Furthermore, they were hospitalized for fewer days, and they never had a feeding problem after the operation. Lastly, the most important two factors, swallowing and communication have never been a problem for this group. The highest score may reflect this general well-being after the operation.

Another interesting outcome of our study is that although the arytenoidectomized patients had longer oral feeding and decanulation times, there are no statistically significant effects on the quality of life, the functional outcomes, or the quality of voice. This suggests that the more laryngeal tissue is removed with the exception of arytenoid, the worse the quality of life, the functional outcomes and the quality of voice will be. This finding further implies that the arytenoid removal does not have an adverse effect on the quality of life, the functional outcomes, or the quality of voice from the patients' point of view. Removing the arytenoid only makes the oral feeding and the decanulation times longer in the first weeks after the operation, but after the tissue heals completely and laryngeal reflexes return to normal, larynx begins compensating arytenoid excision, and functions satisfactorily.

### UW-QOL-R

Since appearance, taste, saliva, chewing and pain are not major issues for most early-stage glottic cancer patients, their discussion was excluded. Swallowing is considered important by the patients, if especially one arytenoid was resected during surgery. Difficulty in swallowing may exist by these patients because of aspiration risk, until the larynx adapts to this new post-surgical situation, and laryngeal reflexes returned to normal. The effect of arytenoidectomy on the swallowing parameter was also analyzed in our study, and interestingly, it is found that the arytenoidectomy has no adverse effect on swallowing after the patients have been discharged.

Since the improvement in swallowing, and the normalcy of voice communication, the two major criteria for quality of life after the laryngeal surgery [7], reflect the level of quality of life in patients treated for laryngeal cancer, it can be suggested again that the [CHP] group had the least favorable quality of life after the operation among the study groups. The significantly longer decanulation and oral feeding times, i.e. longer hospitalization, could be a sign for a relatively worse quality of life.

### PSS-HN

The measures of PSS-HN scored lower for patients who had the [A] procedure. It could be suggested that adding arytenoidectomy would result in lowering of the functional capabilities of the patients, by statistically insignificant amount though.

UOS is strongly related to voice production. The amount of laryngeal tissue excised is highest in patients treated with [CHP] and as expected, the VHI is lowest in these patients. The lowest scores for UOS were given to the [CHP] patients, and as expected, the UOS score is significantly lower in the [CHP] group.

### VHI

Vocal results after [C] and [FLL] could be unsatisfactory, since there is a gap in the creation of a new glottis due to removal of a considerable mass of tissue. After [CHP], the results could be much worse, since both the vocal folds were excised and a neoglottis formation is anticipated. Although statistically insignificant, the [CHP] patient group had given the lowest VHI scores in assessing their new voices in our study. In all of the patient groups, the quality of voice was found to be sufficient to hold a normal individual conversation. However, the voice was defined by the patients as hoarse and dull. It was rated to be insufficient to make a conversation in a noisy atmosphere, since it can not be raised satisfactorily. It also should be remembered that all patients with laryngeal malignancies had voice and communication problems at diagnosis.

Many authors have also studied the quality of voice following radiation therapy for T1 and T2 glottic cancers. Traditionally, radiation therapy has been advocated over surgery by many authors because of the belief that voice quality is superior in the radiation therapy cohort. A review of the literature shows a wide variation in findings [8-15].

The measures VHI questionnaire scored lower for patients who had the [A] procedure. It could be hypothesized that the addition of arytenoidectomy would result in increases in voice handicap of the patients, though statistically insignificant.

## Conclusion

In the present study, all patients with early glottic cancer, treated with different surgical techniques reported fairly good QOL outcomes, functional results and voice qualities. The results of this study also imply that the removal of arytenoid does not have any statistically significant adverse effects on the quality of life, the functional outcomes, or the quality of voice from the patients' point of view.

## Abbreviations

[C] = Cordectomy

[FLL] = Fronto-lateral laryngectomy

[CHP] = Cricohyoidopexi

[A] = Arytenoidectomy

[BFND] = Bilateral functional neck dissection

[QOL] = Quality of life

[UW-QOL-R] = University of Washington- Quality of Life- Revised

[PSS-HN] = Performance Status Scale for Head and Neck cancer patients

[EIP] = Eating in public

[UOS] = Understandability of speech

[NOD] = Normalcy of diet

[VHI] = Voice Handicap Index

[VHI-F] = Voice Handicap Index, functional

[VHI-P] = Voice Handicap Index, physical

[VHI-E] = Voice Handicap Index, emotional

## Competing Interests

The author(s) declare that they have no competing interests.

## Authors' Contributions

TK applied the survey to the patients, assisted in statistical analyses and drafted the manuscript. AS participated in the design of the study. All authors read and approved the final manuscript.

## Pre-publication history

The pre-publication history for this paper can be accessed here:



## Supplementary Material

Additional File 1 Click here for file

## Figures and Tables

**Table 1:** Regression Results. Numbers in parentheses below the coefficients are the t statistics.

**VARIABLE**	**Constant**	**CHP**	**FLL**	**A**	**CHP.A**	**FLL.A**
**Pain**	82.14	-2.14	6.75	9.52	-14.52	1.59
	(8.67)**	(-0.15)	(0.53)	(0.55)	(-0.58)	(0.06)
**Appearance**	89.29	-19.29	-3.17	10.71	10.95	-9.33
	(8.29)**	(-1.16)	(-0.22)	(0.54)	(0.38)	(-0.31)
**Activity**	85.71	-15.71	-2.38	14.29	7.38	-22.62
	(9.04)**	(-1.07)	(-0.19)	(0.82)	(0.29)	(-0.86)
**Recreation**	89.29	-19.29	5.16	10.71	2.62	-55.16
	(10.47)**	(-1.46)	(0.45)	(0.69)	(0.12)	(-2.34) **
**Swallowing**	100.00	-26.60	-14.78	-11.00	26.60	9.28
	(12.80)**	(-2.20)**	(-1.42)	(-0.77)	(1.28)	(0.43)
**Chewing**	100.00	-10.00	-11.11	0.00	10.00	11.11
	(16.53)**	(-1.07)	(-1.38)	(0.00)	(0.62)	(0.67)
**Speech**	89.29	-19.29	-8.73	10.71	2.62	-3.77
	(11.61)**	(-1.62)*	(-0.85)	(0.76)	(0.13)	(-0.18)
**Shoulder**	85.71	-5.51	6.95	3.29	-5.49	-12.45
	(9.34)**	(-0.39)	(0.57)	(0.20)	(-0.22)	(-0.49)
**Taste**	95.29	-8.49	-6.40	-17.62	19.82	28.73
	(11.84)**	(-0.68)	(-0.60)	(-1.20)	(0.93)	(1.29)
**Saliva**	95.29	-8.49	-10.06	-6.29	19.49	21.06
	(13.81)**	(-0.79)	(-1.09)	(-0.50)	(1.06)	(1.11)
**EIP**	100.00	-20.00	-25.00	-33.33	53.33	58.33
	(8.52)**	(-1.10)	(-1.60)	(-1.56)	(1.71)*	(1.80)*
**UOS**	82.14	-27.14	-1.59	-32.14	68.81	39.09
	(11.58)**	(-2.47)**	(-0.17)	(-2.48)**	(3.65)**	(2.00)**
**NOD**	100.00	-4.00	-7.78	-0.00	4.00	7.78
	(21.52)**	(-0.56)	(-1.26)	(-0.00)	(0.32)	(0.61)
**VHI**	69.43	-12.43	-10.54	-6.43	8.10	15.04
	(12.15)**	(-1.40)	(-1.38)	(-0.62)	(0.53)	(0.95)

* denotes significance at 90% confidence level, and ** denotes significance at 95% confidence level.

**Table 2:** The mean values for each analyzed parameter in questionaires, decanulation and oral feeding times for 3 patient group.

	C (n = 10)	FLL (n = 11)	CHP (n = 8)
		***UW-QOL-R part 1***	
Pain	85	91	78
Appearance	92,5	86,3	78,1
Activity	90	81,8	78,1
Recreation	92,5	86,3	75
Swallowing	96,7	84,9	79,3
Chewing	100	90,9	93,8
Speech	92,5	81,8	75
Shoulder	86,7	91	79,4
Taste	90	91	87,6
Saliva	93,4	87,9	91,8
*Total score*	*919,3*	*872,9*	*816,1*
		***UW-QOL-R part 2***	
Question 1	*40*	*13,6*	*12,5*
Question 2	*51,7*	*26,8*	*22,8*
Question 3	*55,1*	*34,5*	*14*
*Total score*	146,8	*74,9*	49,3
		***PSS-HN***	
Eip	90	79,5	87,5
Uos	72,5	81,8	68,8
Nod	100	93,6	97,5
*Total score*	*262,5*	*254,9*	*253,8*
		***VHI***	
VHI	67,5	60,4	57,6
VHI-f	21,8	24	20
VHI-p	22	19,7	17,1
VHI-e	23,7	16,7	20,6
Decanulation (day)	0	3,36	19,25
Oral feeding (day)	1	7,63	22,38

**Table 3:** Number of patient groups and VHI, VHI-F, VHI-P and VHI-E mean values and standart deviations (SD).

GROUP		VHI	VHI-F	VHI-P	VHI-E
C (n = 10)	Mean (SD)	67,50 (17,34)	21,80 (5,49)	22,00 (7,45)	23,70 (9,04)
FLL (n = 11)	Mean (SD)	60,45 (13,97)	24,00 (7,95)	19,73 (6,66)	16,73 (8,58)
CHP (n = 8)	Mean (SD)	57,63 (10,78)	20,00 (4,14)	17,13 (5,46)	20,50 (3,74)

**Table 4:** Decanulation and oral feeding times (day) of the patients and standart deviations (SD).

	Decanulation (day) (SD)	Oral feeding (day) (SD)
C (n = 7)	0	0
C + A (n = 3)	0	1
FLL (n = 9)	2,4 (0,8)	6,2 (1,3)
FLL + A (n = 2)	6,5 (0,7)	13 (1,4)
CHP (n = 5)	18,2 (1,9)	20,6 (2,0)
CHP + A (n = 3)	21,3 (0,5)	24,6 (1,1)
