# Supplementary material for: Quality of life, functional outcome, and voice handicap index in partial laryngectomy patients for early glottic cancer
Source: BMC Ear Nose Throat Disord. 2005 May 12;5:3. doi: 10.1186/1472-6815-5-3 (PMC1142310; doi:10.1186/1472-6815-5-3)
Supplement: Additional File 1 [file 1472-6815-5-3-S1.doc]

| Patient | TNM | Surgery performed | decanulation | oral feeding | pain | appearance | activity | recreation | swallowing | chewing | speech | shoulder | taste | saliva | Global QOL  Question1 | Global QOL  Question2 | Global QOL  Question3 | eip | uos | nod | VHI | VHI-F | VHI-P | VHI-E |
| --- | --- | --- | --- | --- | --- | --- | --- | --- | --- | --- | --- | --- | --- | --- | --- | --- | --- | --- | --- | --- | --- | --- | --- | --- |
| 1 | T1aN0M0 | C+A | 0 | 1 | 100 | 100 | 100 | 100 | 67 | 100 | 100 | 100 | 100 | 100 | 50 | 50 | 67 | 100 | 75 | 100 | 51 | 27 | 14 | 10 |
| 2 | T1aN0M0 | C+A | 0 | 1 | 75 | 100 | 100 | 100 | 100 | 100 | 100 | 67 | 33 | 67 | 25 | 50 | 67 | 0 | 25 | 100 | 68 | 16 | 24 | 28 |
| 3 | T1aN0M0 | C+A | 0 | 1 | 100 | 100 | 100 | 100 | 100 | 100 | 100 | 100 | 100 | 100 | 25 | 50 | 50 | 100 | 50 | 100 | 70 | 23 | 25 | 22 |
| 4 | T1aN0M0 | C | 0 | 1 | 100 | 100 | 100 | 100 | 100 | 100 | 100 | 100 | 100 | 100 | 50 | 67 | 67 | 100 | 75 | 100 | 66 | 22 | 23 | 21 |
| 5 | T1aN0M0 | C | 0 | 1 | 100 | 100 | 100 | 100 | 100 | 100 | 100 | 100 | 100 | 100 | 25 | 50 | 50 | 100 | 75 | 100 | 49 | 17 | 10 | 22 |
| 6 | T1aN0M0 | C | 0 | 1 | 100 | 100 | 75 | 100 | 100 | 100 | 100 | 100 | 100 | 100 | 50 | 50 | 50 | 100 | 75 | 100 | 56 | 16 | 12 | 28 |
| 7 | T1aN0M0 | C | 0 | 1 | 100 | 100 | 75 | 75 | 100 | 100 | 100 | 100 | 100 | 100 | 50 | 50 | 50 | 100 | 100 | 100 | 77 | 22 | 29 | 26 |
| 8 | T1aN0M0 | C | 0 | 1 | 75 | 100 | 100 | 75 | 100 | 100 | 100 | 100 | 100 | 100 | 25 | 50 | 50 | 100 | 75 | 100 | 49 | 16 | 23 | 10 |
| 9 | T1aN0M0 | C | 0 | 1 | 0 | 25 | 50 | 75 | 100 | 100 | 25 | 100 | 100 | 100 | 50 | 50 | 50 | 100 | 75 | 100 | 100 | 30 | 30 | 40 |
| 10 | T1aN0M0 | C | 0 | 1 | 100 | 100 | 100 | 100 | 100 | 100 | 100 | 0 | 67 | 67 | 50 | 50 | 50 | 100 | 100 | 100 | 89 | 29 | 30 | 30 |
| 11 | T1bN0M0 | FLL+Tucker rec+A | 7 | 13 | 100 | 100 | 75 | 100 | 100 | 100 | 100 | 100 | 100 | 100 | 25 | 33 | 50 | 100 | 75 | 100 | 54 | 22 | 14 | 18 |
| 12 | T1bN0M0 | FLL+Tucker rec+A | 6 | 13 | 100 | 75 | 75 | 0 | 67 | 100 | 75 | 67 | 100 | 100 | 25 | 33 | 50 | 100 | 100 | 100 | 81 | 32 | 21 | 28 |
| 13 | T1bN0M0 | FLL+Tucker rec. | 2 | 6 | 100 | 75 | 100 | 100 | 100 | 100 | 75 | 100 | 100 | 100 | 50 | 33 | 50 | 100 | 100 | 100 | 61 | 15 | 34 | 12 |
| 14 | T1bN0M0 | FLL+Tucker rec. | 2 | 7 | 100 | 100 | 100 | 100 | 100 | 100 | 75 | 100 | 100 | 100 | 0 | 33 | 33 | 100 | 100 | 100 | 35 | 13 | 12 | 10 |
| 15 | T1bN0M0 | FLL+Tucker rec. | 3 | 6 | 75 | 100 | 100 | 100 | 100 | 100 | 100 | 67 | 100 | 100 | 0 | 16 | 33 | 100 | 75 | 100 | 45 | 30 | 11 | 4 |
| 16 | T1bN0M0 | FLL+Tucker rec. | 4 | 8 | 100 | 100 | 100 | 100 | 100 | 100 | 100 | 100 | 100 | 100 | 0 | 16 | 16 | 100 | 100 | 100 | 69 | 40 | 21 | 8 |
| 17 | T1bN0M0 | FLL+Tucker rec. | 3 | 6 | 75 | 0 | 25 | 50 | 67 | 50 | 75 | 100 | 67 | 100 | 0 | 16 | 16 | 0 | 75 | 40 | 75 | 26 | 23 | 26 |
| 18 | T1bN0M0 | FLL+Tucker rec. | 2 | 6 | 50 | 100 | 25 | 100 | 33 | 50 | 50 | 100 | 33 | 33 | 25 | 33 | 33 | 25 | 50 | 100 | 70 | 23 | 26 | 21 |
| 19 | T1bN0M0 | FLL+Tucker rec. | 3 | 6 | 100 | 100 | 100 | 100 | 67 | 100 | 100 | 67 | 100 | 100 | 0 | 33 | 33 | 100 | 100 | 100 | 61 | 19 | 18 | 24 |
| 20 | T1bN0M0 | FLL+Tucker rec. | 3 | 7 | 100 | 100 | 100 | 100 | 100 | 100 | 75 | 100 | 100 | 67 | 0 | 33 | 33 | 50 | 50 | 90 | 67 | 26 | 16 | 25 |
| 21 | T1bN0M0 | FLL+Tucker rec. | 2 | 6 | 100 | 100 | 100 | 100 | 100 | 100 | 75 | 100 | 100 | 67 | 25 | 16 | 33 | 100 | 75 | 100 | 47 | 18 | 21 | 8 |
| 22 | T2N0M0 | CHP+BFND+A | 22 | 24 | 50 | 75 | 100 | 75 | 67 | 100 | 75 | 67 | 67 | 100 | 25 | 33 | 16 | 100 | 100 | 100 | 42 | 18 | 11 | 13 |
| 23 | T2N1M0 | CHP+BFND+A | 21 | 24 | 75 | 100 | 100 | 100 | 100 | 100 | 75 | 67 | 100 | 100 | 25 | 33 | 16 | 100 | 100 | 100 | 62 | 21 | 19 | 22 |
| 24 | T2N0M0 | CHP+BFND+A | 20 | 26 | 100 | 100 | 75 | 75 | 100 | 100 | 100 | 100 | 100 | 100 | 25 | 33 | 16 | 100 | 75 | 100 | 72 | 19 | 27 | 26 |
| 25 | T2N0M0 | CHP+BFND | 17 | 20 | 50 | 50 | 25 | 75 | 67 | 50 | 50 | 67 | 67 | 67 | 25 | 16 | 16 | 100 | 50 | 100 | 61 | 23 | 17 | 21 |
| 26 | T2N0M0 | CHP+BFND | 19 | 21 | 75 | 25 | 75 | 25 | 100 | 100 | 50 | 67 | 100 | 100 | 0 | 16 | 0 | 100 | 25 | 100 | 55 | 22 | 11 | 22 |
| 27 | T2N1M0 | CHP+BFND | 18 | 22 | 100 | 100 | 75 | 75 | 33 | 100 | 75 | 100 | 100 | 100 | 0 | 16 | 16 | 50 | 75 | 100 | 61 | 22 | 18 | 21 |
| 28 | T2N0M0 | CHP+BFND | 18 | 22 | 100 | 100 | 100 | 100 | 67 | 100 | 100 | 100 | 67 | 67 | 0 | 16 | 16 | 50 | 50 | 80 | 42 | 11 | 13 | 18 |
| 29 | T2N0M0 | CHP+BFND | 19 | 20 | 75 | 75 | 75 | 75 | 100 | 100 | 75 | 67 | 100 | 100 | 0 | 16 | 16 | 100 | 75 | 100 | 66 | 24 | 21 | 21 |

Additional file 1 : The stages of the laryngeal cancer, the decanulation and the oral feeding times, the surgeries applied, and the scores of the questionaires
